# Supplementary material for: BarlowTwins-CXR: enhancing chest X-ray abnormality localization in heterogeneous data with cross-domain self-supervised learning
Source: BMC Med Inform Decis Mak. 2024 May 16;24:126. doi: 10.1186/s12911-024-02529-9 (PMC11097466; doi:10.1186/s12911-024-02529-9)
Supplement: Supplementary file 1 — Supplementary Material 1. [file 12911_2024_2529_MOESM1_ESM.pdf]

Enhancing Chest X-Ray Based Abnormality Localization with Self-Supervised Learning – Appendices

Weighted box fusion visualization

Figure 1. Weighted box fusion (WBF) visualization preprocessing on the VinDr-CXR dataset - The left column displays original bounding boxes annotated by multiple radiologists, showcasing instances of overlap. The right column demonstrates the outcome after applying WBF, where overlapping annotations are merged into single, fused bounding boxes, thus enhancing the precision of the target area representation.

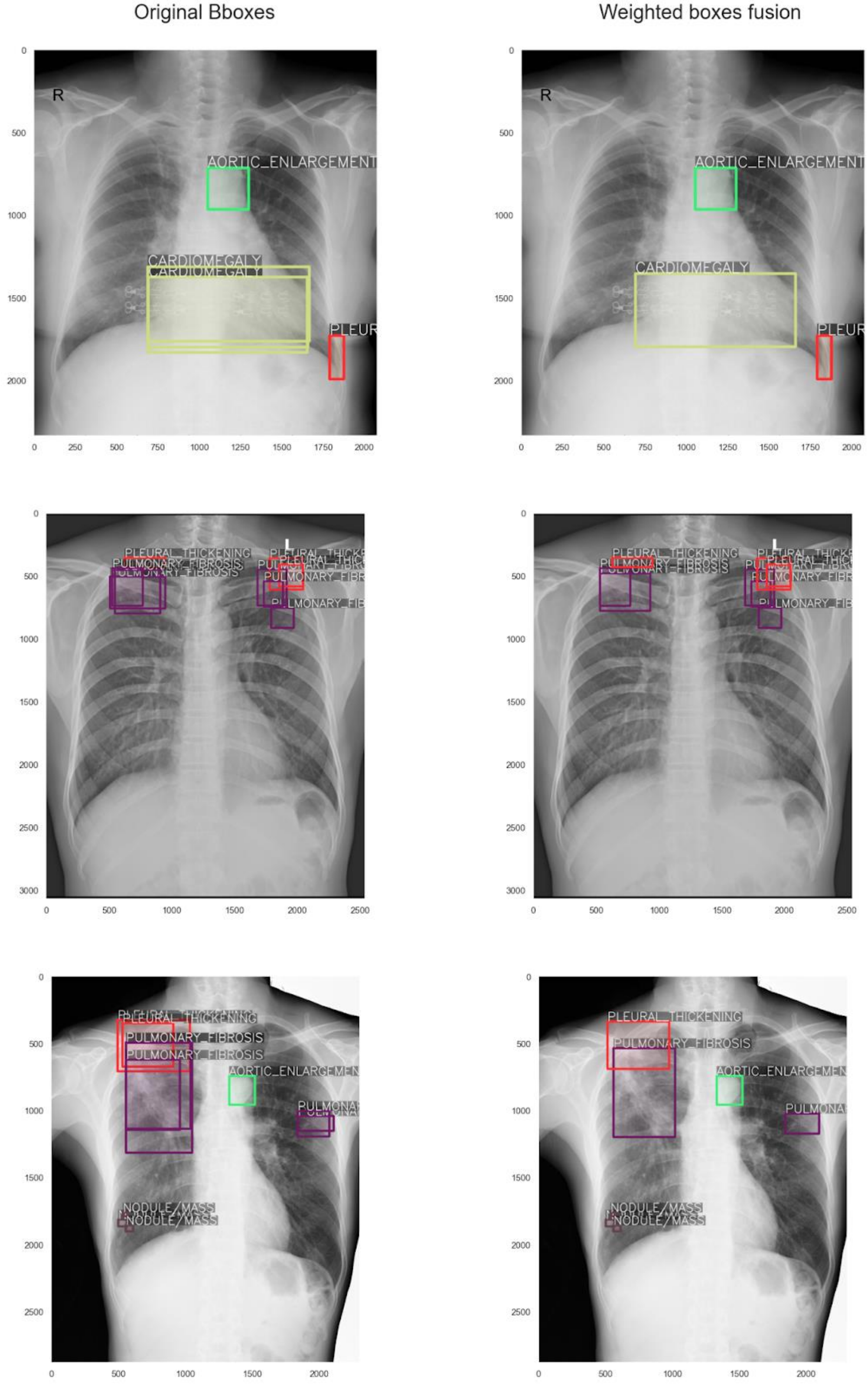

Individually labeled linear evaluation protocol results

Table 1. The appended table comprehensively summarizes the Area Under the Curve (AUC) scores for each condition when applying the linear evaluation protocol to the NIH-CXR dataset. Scores are presented for models trained with 100%, 10%, and 1% of the available labelled data. For each condition, the AUC scores obtained from models pre-trained with Barlow Twins-CXR are compared against those pre-trained with ImageNet, with the difference between them indicated in parentheses. The average AUC scores across all conditions are also provided for an overall performance overview. These evaluations were consistently generated using a random seed of 42 to ensure reproducibility.

**Condition**	**lincls 100<br>(Barlow, ImageNet, Difference)**	**lincls 10**	**lincls 1**
**Atelectasis**	0.764, 0.674(0.090)	0.738, 0.644(0.094)	0.613, 0.533(0.080)
**Consolidation**	0.726, 0.669(0.057)	0.708, 0.659(0.049)	0.595, 0.527(0.068)
**Infiltration**	0.677, 0.650(0.027)	0.672, 0.637(0.035)	0.559, 0.597(-0.038)
**Pneumothorax**	0.871, 0.776(0.095)	0.854, 0.754(0.100)	0.760, 0.686(0.074)
**Edema**	0.860, 0.772(0.088)	0.858, 0.761(0.097)	0.657, 0.582(0.075)
**Emphysema**	0.864, 0.790(0.074)	0.816, 0.758(0.058)	0.739, 0.690(0.049)
**Fibrosis**	0.862, 0.755(0.107)	0.801, 0.668(0.133)	0.609, 0.671(-0.062)
**Effusion**	0.803, 0.727(0.076)	0.794, 0.700(0.094)	0.693, 0.602(0.091)
**Pneumonia**	0.664, 0.624(0.040)	0.608, 0.568(0.040)	0.584, 0.507(0.077)
**Pleural Thickening**	0.796, 0.653(0.143)	0.769, 0.623(0.146)	0.621, 0.589(0.032)
**Cardiomegaly**	0.908, 0.757(0.151)	0.897, 0.721(0.176)	0.790, 0.567(0.223)
**Nodule**	0.771, 0.653(0.118)	0.728, 0.608(0.120)	0.610, 0.558(0.052)
**Mass**	0.819, 0.618(0.201)	0.816, 0.608(0.208)	0.606, 0.562(0.044)
**Hernia**	0.972, 0.907(0.065)	0.943, 0.864(0.079)	0.809, 0.599(0.210)
**No Finding**	0.683, 0.623(0.060)	0.681, 0.614(0.067)	0.604, 0.579(0.025)
**Average AUC**	0.803, 0.710(0.093)	0.779, 0.679(0.100)	0.657, 0.590(0.067)

Individually labeled End-to-End Fine-Tuning results

Table 2. Detailed Label Performance in End-to-End Fine-Tuning Evaluation Results

The following table in the appendix delineates the outcomes of our End-to-End fine-tuning evaluation protocol on the NIH-CXR dataset. Scores are presented for models trained with 100%, 10%, and 1% of the available labelled data. For each condition, AUC scores obtained from models pre-trained with Barlow Twins-CXR are compared against those pre-trained with ImageNet, with the difference between them indicated in parentheses. The average AUC scores across all conditions are also provided for an overall performance overview. These evaluations were consistently generated using a random seed of 42 to ensure reproducibility.

**Condition**	**finetuning 100**<br>**(Barlow, ImageNet, Difference)**	**finetuning 10**	**finetuning 1**
**Atelectasis**	0.775, 0.760(0.015)	0.748, 0.708(0.040)	0.629, 0.580(0.049)
**Consolidation**	0.747, 0.733(0.014)	0.711, 0.690(0.021)	0.656, 0.611(0.045)
**Infiltration**	0.671, 0.660(0.011)	0.676, 0.638(0.038)	0.560, 0.592(-0.032)
**Pneumothorax**	0.892, 0.857(0.035)	0.855, 0.799(0.056)	0.749, 0.644(0.105)
**Edema**	0.856, 0.851(0.005)	0.835, 0.788(0.047)	0.684, 0.663(0.021)
**Emphysema**	0.887, 0.877(0.010)	0.817, 0.790(0.027)	0.699, 0.609(0.090)
**Fibrosis**	0.874, 0.826(0.048)	0.801, 0.663(0.138)	0.730, 0.713(0.017)
**Effusion**	0.813, 0.794(0.019)	0.798, 0.755(0.043)	0.680, 0.636(0.044)
**Pneumonia**	0.668, 0.628(0.040)	0.592, 0.618(-0.026)	0.549, 0.491(0.058)
**Pleural Thickening**	0.799, 0.751(0.048)	0.757, 0.664(0.093)	0.650, 0.651(-0.001)
**Cardiomegaly**	0.903, 0.885(0.018)	0.886, 0.818(0.068)	0.808, 0.634(0.174)
**Nodule**	0.787, 0.764(0.023)	0.728, 0.610(0.118)	0.618, 0.608(0.010)
**Mass**	0.836, 0.791(0.045)	0.814, 0.664(0.150)	0.613, 0.535(0.078)
**Hernia**	0.973, 0.899(0.074)	0.929, 0.863(0.066)	0.704, 0.688(0.016)
**No Finding**	0.698, 0.680(0.018)	0.682, 0.643(0.039)	0.612, 0.564(0.048)
**Average AUC**	0.812, 0.784(0.028)	0.775, 0.714(0.061)	0.663, 0.615(0.048)

Confusion matrix of the VinDr-CXR test set

Figure 2: Normalized Confusion Matrices for Barlow Twins-CXR and ImageNet Pre-trained Backbones on the VinDr-CXR Dataset

The confusion matrices presented here showcase the predictive outcomes on the VinDr-CXR test set following training with two different pre-trained backbones: Barlow Twins-CXR(a) and ImageNet(b), both utilizing a 224x224 input resolution. The diagonal values represent the proportion of correctly predicted bounding boxes per category. Notably, despite the challenges of sparse data and imbalanced distribution, which negatively impacted the ImageNet backbone's performance for certain classes, the Barlow Twins-CXR approach somewhat mitigates these issues, suggesting its robustness in dealing with class imbalance and data scarcity.

a)

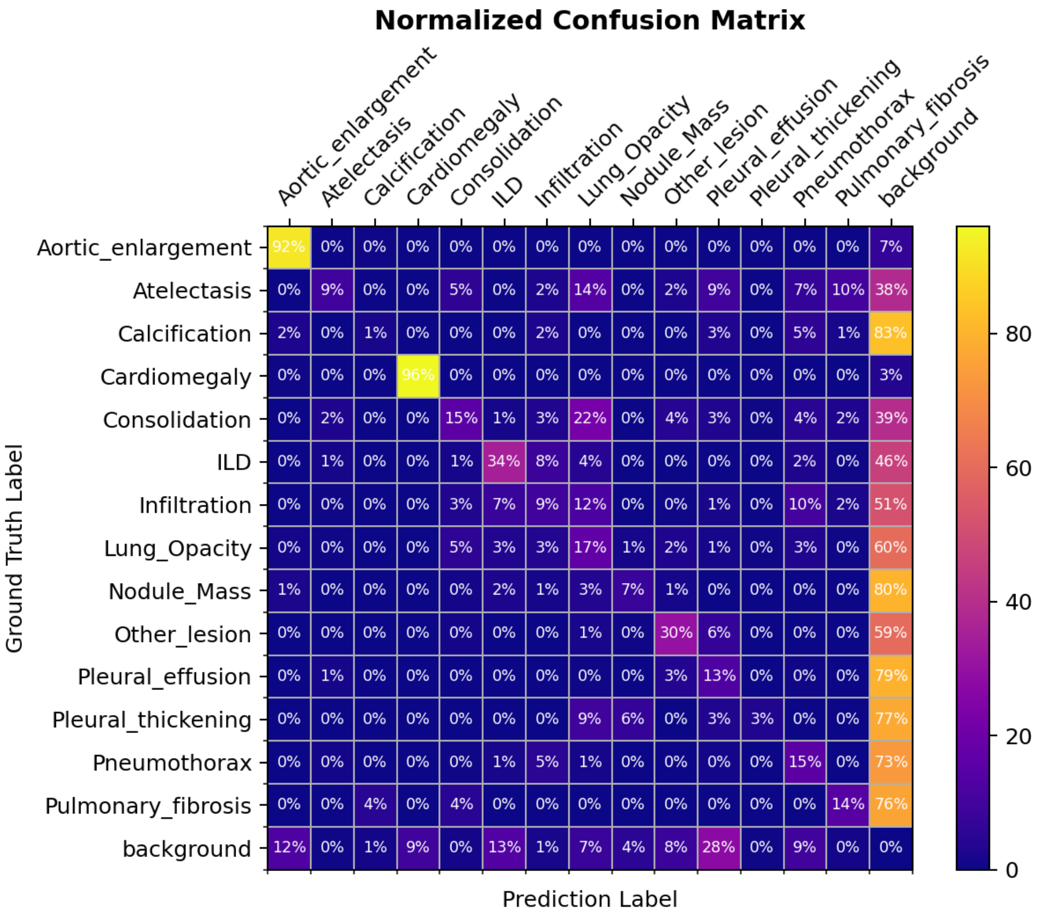

b)

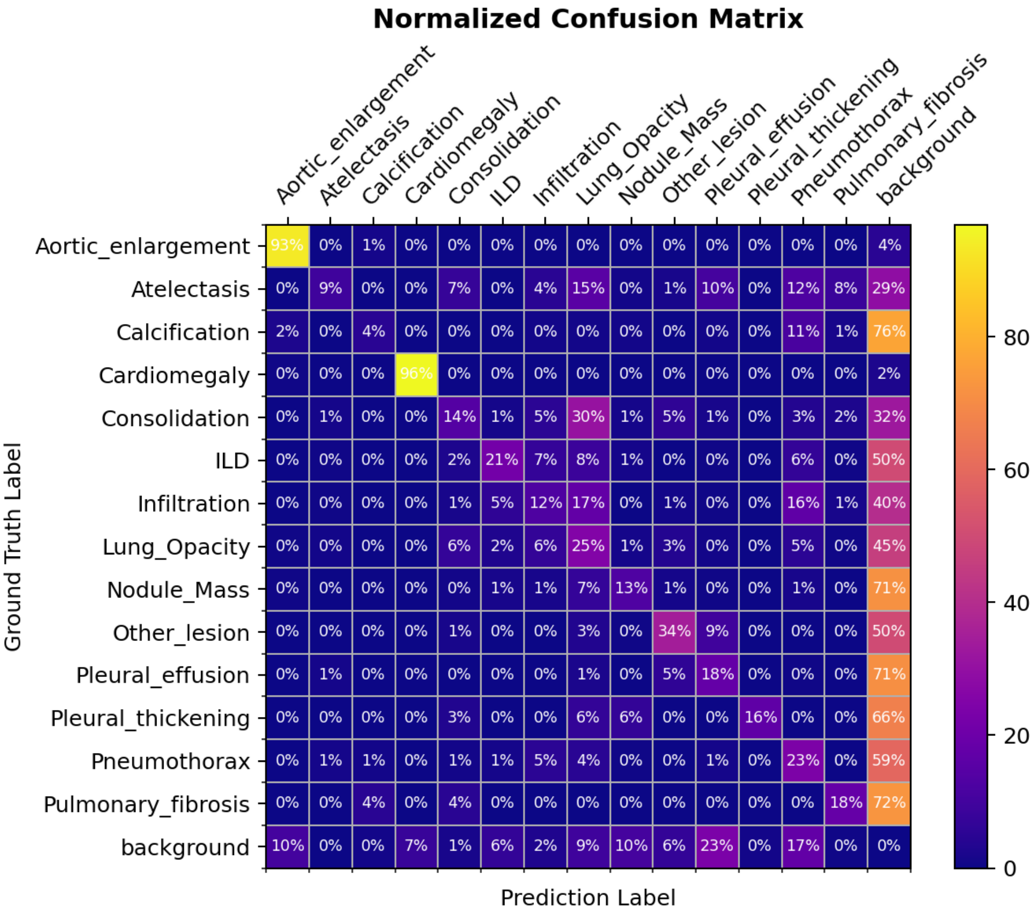
Normalized Confusion Matrix
Aortic_enlargement
Atelectasis
Calcification
Cardiomegaly
Consolidation
ILD
Infiltration
Lung_Opacity
Nodule_Mass
Other_lesion
Pleural_effusion
Pleural_thickening
Pneumothorax
Pulmonary_fibrosis
background
Aortic_enlargement 93% 0% 1% 0% 0% 0% 0% 0% 0% 0% 0% 0% 0% 0% 4%
Atelectasis 0% 9% 0% 0% 7% 0% 4% 15% 0% 1% 10% 0% 12% 8% 29%
Calcification 2% 0% 4% 0% 0% 0% 0% 0% 0% 0% 0% 0% 11% 1% 76%
Cardiomegaly 0% 0% 0% 96% 0% 0% 0% 0% 0% 0% 0% 0% 0% 0% 2%
Consolidation 0% 1% 0% 0% 14% 1% 5% 30% 1% 5% 1% 0% 3% 2% 32%
ILD 0% 0% 0% 0% 2% 21% 7% 8% 1% 0% 0% 0% 6% 0% 50%
Infiltration 0% 0% 0% 0% 1% 5% 12% 17% 0% 1% 0% 0% 16% 1% 40%
Lung_Opacity 0% 0% 0% 0% 6% 2% 6% 25% 1% 3% 0% 0% 5% 0% 45%
Nodule_Mass 0% 0% 0% 0% 0% 1% 1% 7% 13% 1% 0% 0% 1% 0% 71%
Other_lesion 0% 0% 0% 0% 1% 0% 0% 3% 0% 34% 9% 0% 0% 0% 50%
Pleural_effusion 0% 1% 0% 0% 0% 0% 0% 1% 0% 5% 18% 0% 0% 0% 71%
Pleural_thickening 0% 0% 0% 0% 3% 0% 0% 6% 6% 0% 0% 16% 0% 0% 66%
Pneumothorax 0% 1% 1% 0% 1% 1% 5% 4% 0% 0% 1% 0% 23% 0% 59%
Pulmonary_fibrosis 0% 0% 4% 0% 4% 0% 0% 0% 0% 0% 0% 0% 0% 18% 72%
background 10% 0% 0% 7% 1% 6% 2% 9% 10% 6% 23% 0% 17% 0% 0%
Ground Truth Label
Prediction Label
80
60
40
20
0

Ablation experiment - Linear Evaluation Protocol

We set up ablation experiments to demonstrate the performance of Barlow Twins-CXR under different hyperparameter settings.

Effect of the dimensionality of the layer of the projector network on performance. For the results of Linear Evaluation Protocol, Barlow Twins-CXR performs better when the dimension of the output of the projection network increases (Figure3 and Table2). This finding was also confirmed in the original Barlow twins paper.

Figure 3: AUC Scores for NIH-CXR Classification - This figure displays the AUC scores of linear models with Barlow Twins-CXR with different projector width hyperparameter across various dataset sizes (1%, 10%, 100%). As indicated by higher AUC scores, models with larger projector widths result in higher performance.

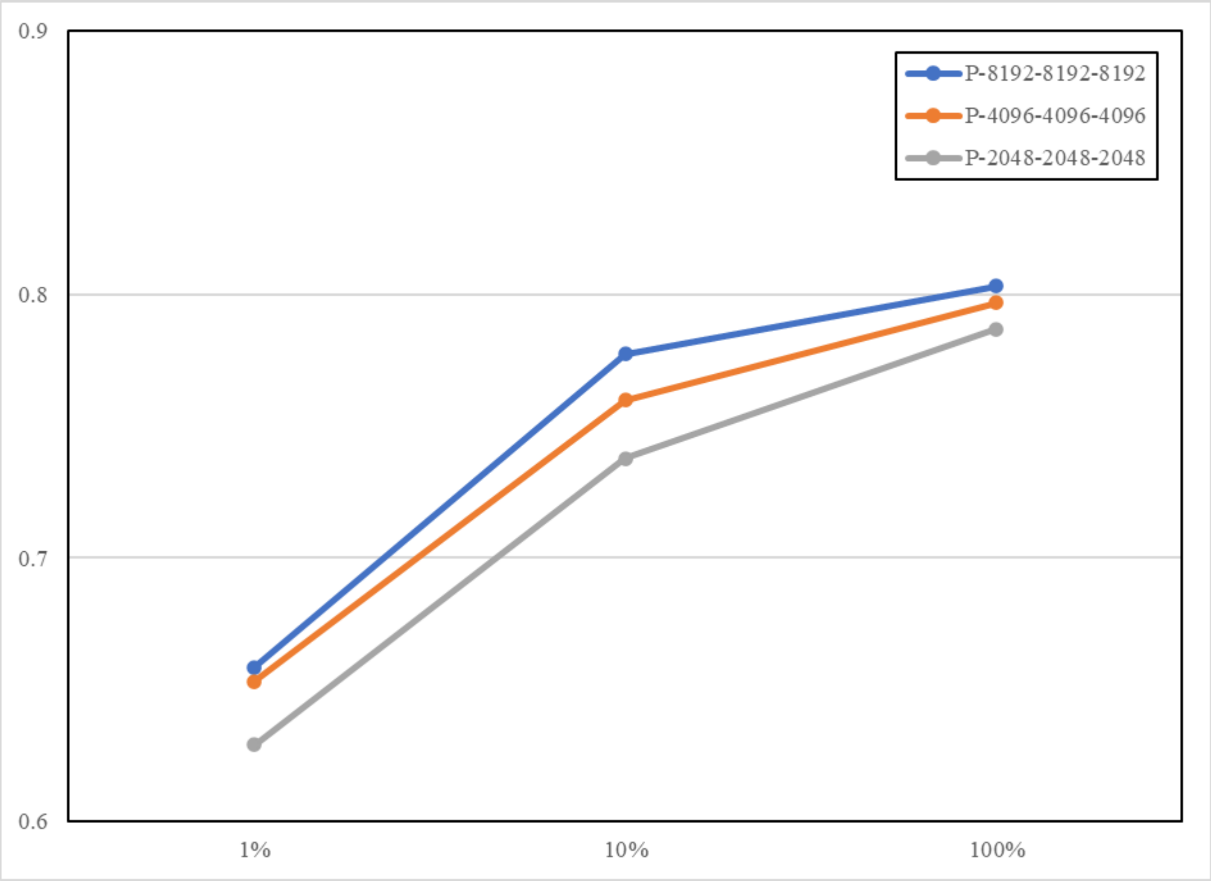

Table 2. AUC scores in validation and test sets for of linear models with varying projector widths.

**Projector**	**1%**	**10%**	**100%**
8192-8192-8192	0.6586	0.7773	0.8031
4096-4096-4096	0.6531	0.7600	0.7969
2048-2048-2048	0.6292	0.7377	0.7869

Effect of the λ on performance, which is used to trade off the invariance and informativeness of the embeddings. For the results of Linear Evaluation Protocol, Barlow Twins-CXR does not change significantly for different λ settings and is therefore insensitive to this hyperparameter (Figure4 and Table3). This is also confirmed in the original Barlow twins paper.

Figure 4: AUC Scores for NIH-CXR Classification - This figure displays the AUC scores of linear models with Barlow Twins-CXR with different λ hyperparameter across various dataset sizes (1%, 10%, 100%). As indicated by the nearly identical AUC scores, models are not sensitive to changes of λ.

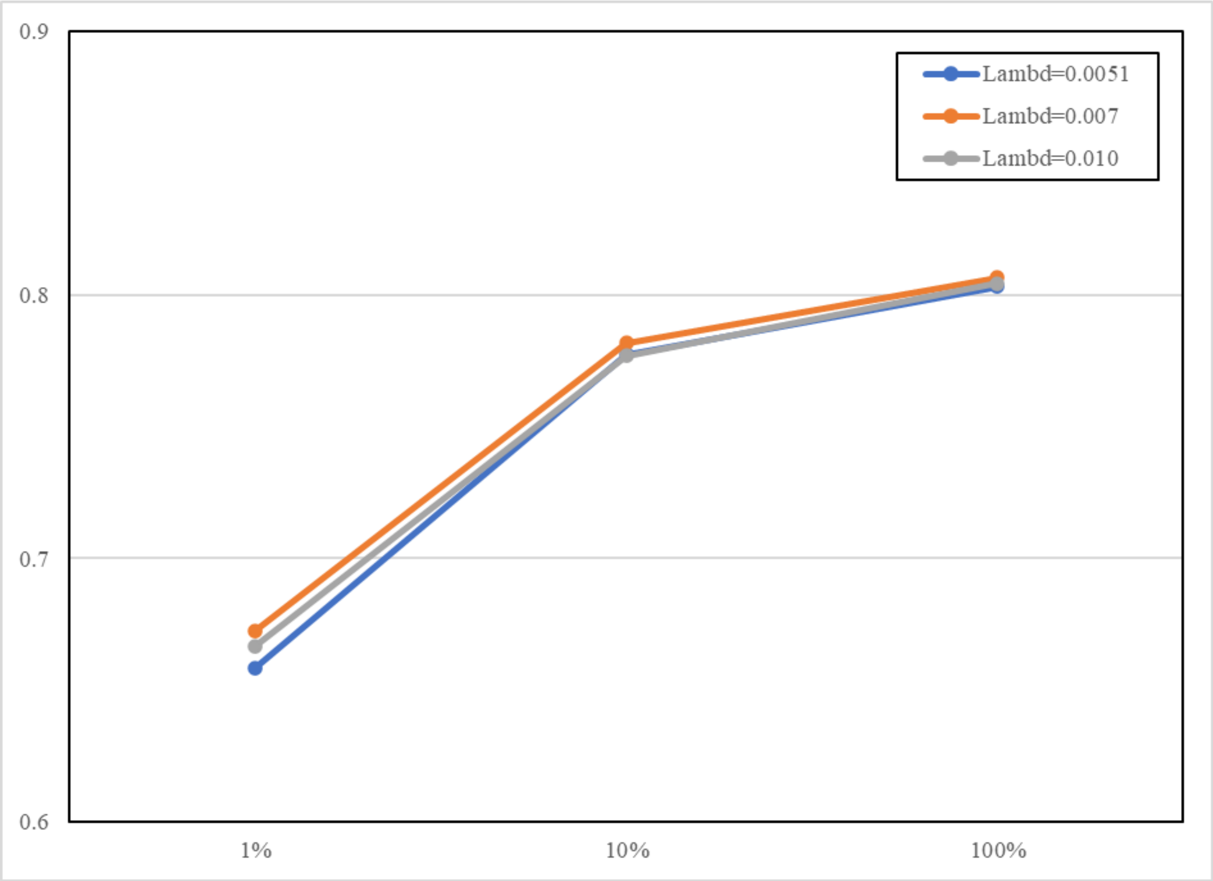

Table 3. AUC scores in validation and test sets for of linear models with varying with different λ setting.

**λ**	**1%**	**10%**	**100%**
0.0051	0.6586	0.7773	0.8031
0.007	0.6726	0.7819	0.8066
0.010	0.6669	0.7770	0.8043

Ablation experiment – Transfer Learning on VinDr Abnormality Localization

In the VinDr anomaly localization downstream tasks, we noticed that the settings of different hyperparameters had no significant impact on the final results.

This proves that for this specific downstream task, the Barlowtwins-CXR method can improve performance while being insensitive to the two hyperparameters of projector width and λ.

The results of the ablation study demonstrate that the Barlowtwins-CXR method can enhance performance for this particular downstream task, while remaining largely unaffected by the two hyperparameters of projector width and λ.

Table 4. mAP50 scores in validation and test sets for models with varying pre-training hyperparameter setting at 224 input resolutions.

**Projector**	**λ**	**mAP50(val set)**	**mAP50(test set)**
8192-8192-8192	0.0051	0.262(0.256,0.268)	0.250(0.247 0.252)
4096-4096-4096		0.264(0.258,0.270)	0.253(0.248,0.258)
2048-2048-2048		0.269(0.262,0.277)	0.251(0.246,0.257)
8192-8192-8192	0.007	0.271(0.266,0.278)	0.258(0.247,0.269)
	0.010	0.268(0.264,0.273)	0.253(0.243,0.263)
